# Low-Profile Slotted Metamaterial Antenna Based on Bi Slot Microstrip Patch for 5G Application

**DOI:** 10.3390/s20113323

**Published:** 2020-06-11

**Authors:** Ahasanul Hoque, Mohammad Tariqul Islam, Ali F. Almutairi

**Affiliations:** 1 Department of Electrical, Electronic and Systems Engineering, Faculty of Engineering and Built Environment, Universiti Kebangsaan Malaysia, Bangi 43600, Selangor, Malaysia; p94155@siswa.ukm.edu.my; 2 Electrical Engineering Department, Kuwait University, Kuwait City 13060, Kuwait

**Keywords:** antenna, metamaterial, low profile, slotted, 5G

## Abstract

A low-profile high-directivity, and double-negative (DNG) metamaterial-loaded antenna with a slotted patch is proposed for the 5G application. The radiated slotted arm as a V shape has been extended to provide a low-profile feature with a two-isometric view square patch structure, which accelerates the electromagnetic (EM) resonance. Besides, the tapered patch with two vertically split parabolic horns and the unit cell metamaterial expedite achieve more directive radiation. Two adjacent splits with meta units enhance the surface current to modify the actual electric current, which is induced by a substrate-isolated EM field. As a result, the slotted antenna shows a 7.14 dBi realized gain with 80% radiation efficiency, which is quite significant. The operation bandwidth is 4.27–4.40 GHz, and characteristic impedance approximately remains the same (50 Ω) to give a VSWR (voltage Standing wave ratio) of less than 2, which is ideal for the expected application field. The overall size of the antenna is 60 × 40 × 1.52 mm. Hence, it has potential for future 5G applications, like Internet of Things (IoT), healthcare systems, smart homes, etc.

## 1. Introduction

Mobile or wireless technology is rapidly changing its high-speed data transfer requirement strategy, thus pushing researchers to develop a faster and dynamic system of communication devices. Increasingly more users are coming online, making existing fourth-generation (4G) technology approach capacity, whereas the demand is increasing. Now, fifth generation (5G) mobile technology is being developed to fulfil this requirement for various applications. Hence, different radio wavebands from 3–5 GHz are allotted to Europe, the USA, and China. Enormous amounts of 5G applications demand versatile properties in antenna parameters, such as a stable high gain and radiation pattern, good beam focusing, and directivity. In the last few years, negative index materials (NIMs), also known as metamaterial, have been widely mentioned in reported articles to expedite the versatility of 5G antennas, such as the frequency selectivity, beam steering, gain, and bandwidth enhancement [1,2,3,4,5]. A balanced resonator structure using metamaterial has wide applicability within antenna applications. For instance, microwave image processing [6,7,8], fluid sensing [9], biomedical or diagonalization research [10,11], electromagnetic (EM) cloaking and Specific Absorption Rate (SAR) reduction [12,13], etc. All these studies have shown that the metamaterial behaves well with the antenna. However, few studies have focused on out-of-band rejection and its practical applications. With the proposal of fifth-generation wireless systems, people have paid more attention to the use of the necessary band and good rejection outside the working band. Some new antennas have been designed for good out-of-band rejection, but the whole structure of these antennas is larger. Some antennas have a dual-polarization characteristic but do not behave well in the out-of-band rejection. Moreover, other filtering antennas with the multilayer coupled radiation structure have been designed and possess an out-of-band rejection function; however, the high selective filtering property of the antenna needs to be improved [14].

Numerous communication system and IoT applications in 5G are not just an evolutionary upgrade of the conventional cellular systems but are envisioned to improve quality of life by encompassing massive connectivity for new mobile application, e.g., in telemedicine, eHealth, machine-to-machine (M2M) communication, autonomous vehicles, and smart cities and homes. More recently, the low-profile antenna with high gain and directivity has been a crucial task for antenna developers. There are only a few types of antennas that are promising for such applications. An H-plane ridged substrate-integrated waveguide (SIW) horn antenna mounted on a large ground plane was introduced [15]. This antenna has an arc-shaped horn aperture with the wideband operation, but due to cone-shaped feeding, it is more difficult to fabricate. The low-profile surface wave antenna developed by Chen and Shen has a grounded ceramic slab with a very high dielectric constant (ε_r_ = 25) [16]. Besides, it was shown to achieve a wide bandwidth and a stable and quasi end-fire radiation beam for potential applications. The log-periodic array antenna is also beneficial since a wide bandwidth is easily achieved for the large metallic platform in 5G applications. Hu and his research group demonstrated a unique low-profile log-periodic monopole array antenna, where top-hat loading of the patch structure exemplified a height reduction benefit [17]. A very low-profile antenna of 0.053 λ_L_ (free space wavelength) was reported, where a wide bandwidth was attained with lower reflected power from the antenna [18]. Artificial magnetic conductor (AMC) surface-based low-profile antenna also achieved a narrow bandwidth with a two-dimensional array. AMC shows in-phase reflection characteristics in a particular frequency spectrum, which expands its applicability as a reflector antenna [19,20,21,22]. Earlier than these stated articles, metamaterial resonators of various shapes were used for low-profile dipole antennas. For example, dogbone-shaped metallic conductors were implemented in a dipole antenna to enhance symmetric and antisymmetric resonance. Furthermore, antisymmetric resonance comes along with artificial magnetism, which helps to reduce the prototype antenna’s thickness [23,24,25,26,27].

The slotted antenna, or the extended arm Vivaldi metamaterial antenna (VMA), has a unique feature of EM wave propagation modification. This particular shape was first introduced by Gibson [28], where a sharp coincident of the adjacent patch creates a significant concentration of the EM field. Besides, the slot lines use a specific radiation mechanism to maintain a strong feeding continuity and radiating ranges. It is noteworthy to mention that the bandwidth and wideband operation depend on the microstrip patch dimension. Recent stated [29,30,31,32] antennas used an array combination to achieve versatile characteristics.

In this paper, a high-directivity double-negative (DNG) metamaterial-loaded antenna is reported. The balanced positioning of the DNG unit cell at both the front and backplane of the antenna harmonizes the electromagnetic (EM) field distribution. Besides, the retro shape of the slotted antenna and consecutive DNG cell achieve high reflection. The main goal was to maintain the specific bandwidth from 4.12–4.44 GHz with an average gain of 6.65 dBi both in the simulated and measured case. The realized antenna was first designed, simulated, and optimized in commercially available Computer Simulation Technology (CST) microwave studio 2017, followed by the Ansoft 3D electromagnetic high-frequency simulator (HFSS), and then validated by fabrication and measurement. Thus, the DNG feature and extended slotted arm patch expedite a particular narrowband operation. The analytical and measured performance ensure the proposed antenna can be a good candidate for sub-6 GHz potential applications.

## 2. Antenna Design and Methodology

### 2.1. Antenna Geometry

**Figure 1:**
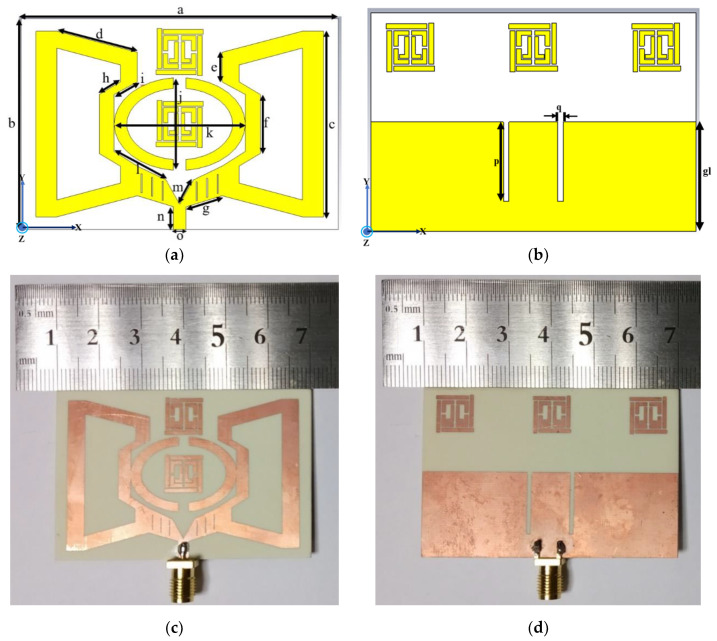
Proposed VMA design geometry (**a**) front view (**b**) bottom view; fabricated front (**c**) and bottom (**d**).

The proposed antenna, as shown in Figure 1, illustrates a narrowband operation loaded with a metamaterial unit. The structure of the slotted metamaterial antenna contains a 60 × 40 mm^2^ unit cell antenna, and the feedline was set to 4.32 mm with a width of 2.40 mm to achieve 50 Ω impedance. The physical architecture of design and fabricated retro VMA was used on Rogers RO4350B epoxy PTFE-based materials with a thickness of 1.52 mm. This material maintains a low loss tangent of 0.0037 and relative permittivity (ε_r_) of 3.48. The electrical dimension was 0.58 λ × 0.30 λ × 0.010 λ considering the lowest −10 dB at the resonance frequency. The patch structure clearly shows an extended V-shaped arm, creating balance in the EM field resonance. Besides, a significant amount of surface current flows in each part, which will be discussed in the following sections. Similarly, the ground plane comprises of the half ground patch with two slits near the feedline and three DNG meta unit cells, ensuring the balance in the patch structure feeding and surface current control. A tapered V-shaped arm with an extended patch excites the antenna and steers between the impedance and feed.

In each arm, there are three 3.6 × 0.2-mm-long slits and one parabolic horn adjoined to the tapered arm with an optimized gap of 2.40 mm. These slits and horns concentrate the electric field at a particular frequency to achieve a narrowband operation in the desired sub-6 GHz band. Hence, the optimized physical parameters of the proposed retro VMA are given in Table 1.

### 2.2. Transmission Line Principle Analysis of VMA Geometry

**Figure 2:**
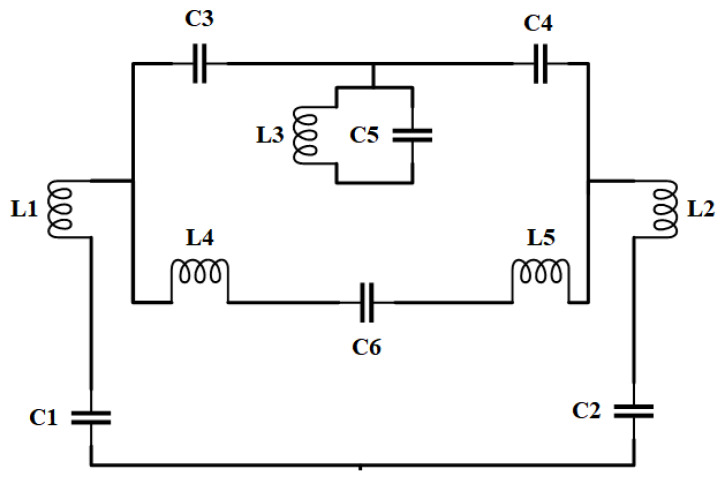
Equivalent circuit of the proposed retro VMA using the transmission line principle.

To obtain the retro VMA design for anticipated band operation, we first analyzed the transmission line principle [33,34] with improved accuracy for the narrow patch [35]. Generally, the edge-coupled microstrip feed calculated using step-in-width/impedance junction suffers from an impedance mismatch. Besides, the gap-coupled feed and coplanar microstrip feed each has limitations regarding the power handling capability and spurious radiation. There is another feeding technique available [33]; rather, we chose to model and analyze the patch structure using the transmission line model. This model improves three major aspects in radiative coupling, side slot radiation conductance, and convenient analytical expression, thus making it more convenient for any microstrip structure. Despite such a significant advantage of the transmission line method, the results regarding the accuracy and versatility of the application have limitations. However, the equivalent circuit, as shown in Figure 2, was approximated following the equation:

(1) $$f_{r}=\frac{1}{2\pi \sqrt{L_{eq}C_{eq}}},$$

where *f_r_* = resonance frequency of operation, *L_eq_* = equivalent inductance, and *C_eq_* = equivalent capacitance. For low-frequency operation, the effective dielectric constant (*ε_reff_*) is:

(2) $$\varepsilon_{reff}=\frac{\varepsilon_{r}+1}{2}+\frac{\varepsilon_{r}-1}{2}{\left[1+12\frac{h}{w}\right]}^{-1/2}\;(\mathrm{for}\;w/h>1),$$

(3) $$w=\frac{\nu_{0}}{2f_{r}\sqrt{\frac{\varepsilon_{r}+1}{2}}},$$

(4) $$L=\frac{\nu_{0}}{2f_{r}\sqrt{\varepsilon_{reff}}}-0.824h\left(\frac{\left(\varepsilon_{reff}+0.3)(\frac{w}{h}+0.264\right)}{\left(\varepsilon_{reff}-0.258)(\frac{w}{h}+0.8\right)}\right),$$

where *w* = width of microstrip patch, *h* = height of substrate, and *υ*_0_ = velocity of light in free space. They are subject to fringing effects, which means the patch of the microstrip antenna looks electrically greater than its physical dimensions. It is noteworthy to mention that an increase of the substrate height expedites fringing and leads to larger separations between the feedline and resonance frequency. However, inductors (L1 to L6) and capacitors (C1 to C6) individually signify each microstrip on the proposed antenna. *L_eq_* and *C_eq_* are calculated using Equations (3) and (4) using the lumped components’ characteristics [36,37].

### 2.3. Metamaterial DNG Unit Cell Design Development and Refinement

**Figure 3:**
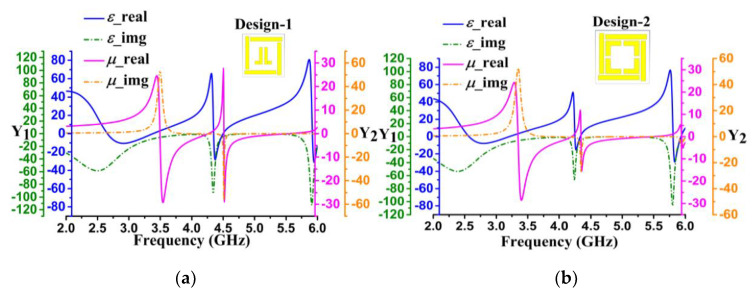

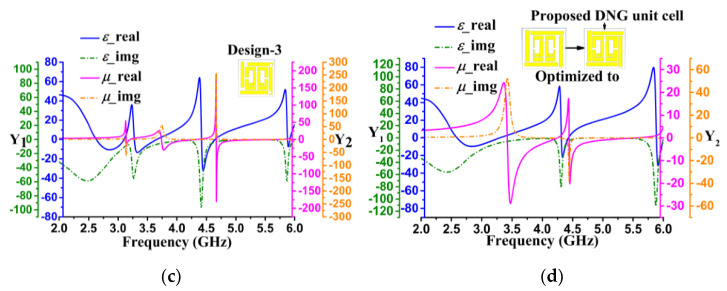
Metamaterial DNG unit cell geometry evolution and dielectric performance in the simulation for (**a**) Design-1, (**b**) Design-2, (**c**) Design-3, and (**d**) the optimized DNG unit cell. Y_1_ and Y_2_ respectively represent the two vertical axes for permittivity and permeability.

Metamaterial DNG unit cell embedded in the proposed retro VMA is a design based on the finite integration technique (FIT) and detailed design procedure reported in the article [38]. This split pitch square (SPS) design forms a perfect resonance circuit using the microstrip patch; thus, the EM signal is stuck between two vertically balanced sections. Moreover, impedance and admittance have been calculated through proper stimulation to obtain negative index metamaterial properties, widely termed as DNG. Figure 3 illustrates the overall design evolution with dielectric parameters (*ε*, *µ*) for DNG characteristics’ existence in the operational bandwidth numerically. The initial unit cell design, design-1 (Figure 3a), started with the SPS patch near the structure edge and the mirror-reflexed L shape at the center with a gap of 0.5 mm. The negative index of *ε* = 3.4 starts at approximately 4.32 GHz and continues up to −26.5, though *µ* starts to show a positive index of 1.64. Unfortunately, at 4.27 GHz, *µ* is only −0.62, which implies that design-1 has a DNG feature but is not so significant in the entire operational bandwidth. Followed by design-2 and design-3 in Figure 3b,c, both parameters try to dominate each other, specially design-3. Design-2 includes an additional square patch without center patching, whereas design-3 comprises the L shape patch. Thus, the mutual resonance expedites but shifts the permittivity (−8.15, at 4.4 GHz) and permeability (−0.81, at 4.29 GHz) at the edge of the bandwidth in design-3. Besides both designs, the performance variation indicates combining and optimizing the patch gaps and widths would enhance the possibility of obtaining a more negative index simultaneously. Finally, in Figure 3d, optimizing the square patch width horizontally obtains the metamaterial DNG unit cell’s geometry. Notably, the waveguide port configuration achieves *ε* = −16.2 and *µ* = −0.55 at 4.30 GHz. Therefore, the floquet port excitation technique was further investigated numerically to verify the DNG characteristics.

**Figure 4:**
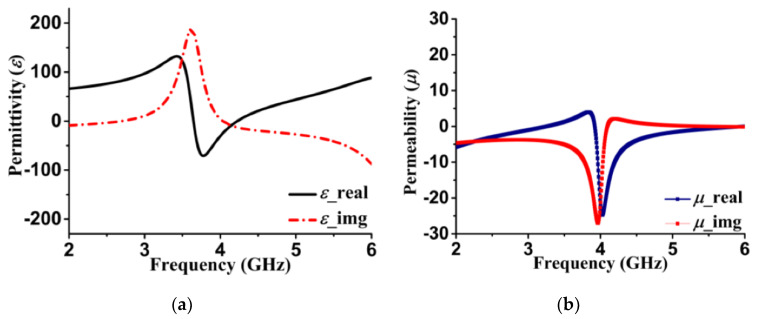
DNG metamaterial dielectric properties in simulation: (**a**) Permittivity (ε) and (**b**) permeability (µ).

The waveguide port configuration reveals the X band performance of SPS [38], but to validate the negative permittivity (*ε*) and permeability (*µ*) feature for the proposed antenna, CST’s default Floquet theorem was adopted. The theorem considers the SPS as a periodic structure, and periodic boundary conditions for theta (θ), and phi (φ) accordingly. The Eigenmode solver was applied particularly for TE_10_ mode and the corresponding dielectric properties (*ε*, *µ*) are plotted in Figure 4. The simulated data illustrate that the real value of *ε* is approximately −1.1, and the real *µ* is −9.4 at 4.27 GHz. Following the bandwidth (up to 4.4 GHz), the permeability remains negative (−4.94), but unfortunately, the permittivity becomes positive. Thus, DNG characteristics exist between 4.27 and 4.4 GHz and indicate a potentiality to enhance the performance based on the literature study. However, the number of the unit cell for the front and ground plane identified through the extensive parametric study will be described in the following sections.

### 2.4. Parametric Study of Antenna

**Figure 5:**
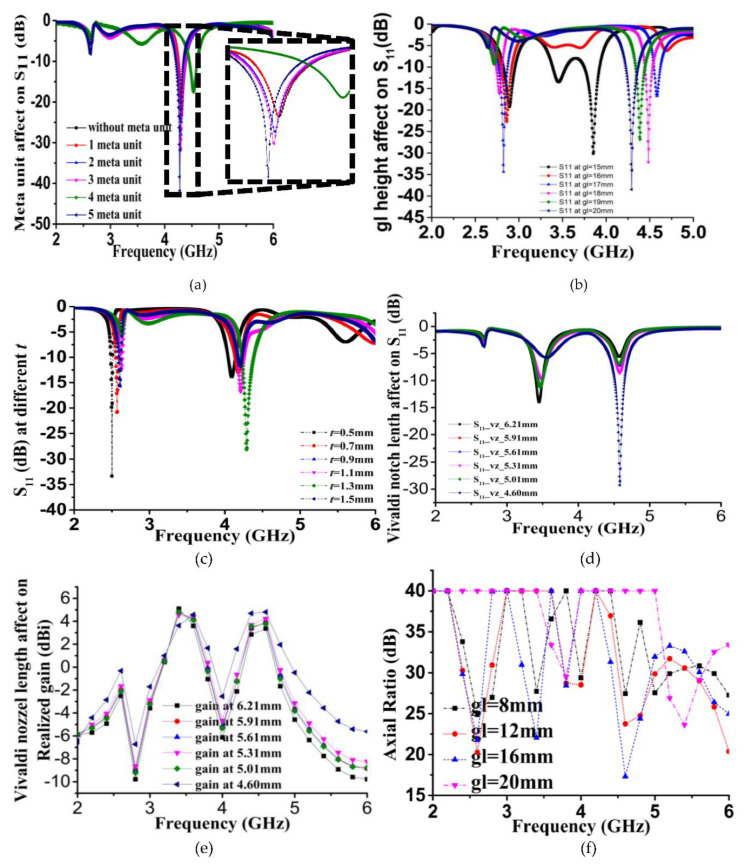
Performance analysis using parametric variation: (**a**) Number of metamaterial unit cell variation and (**b**) ground plane height affects (**c**) substrate thickness, (***t***) variation; (**d**) slotted notch length effect on the reflection coefficient (S_11_) (**e**) realized gain vs. slotted notch variation; (**f**) axial ratio vs. ground plane height variation.

The proposed retro VMA with an extended V arm and metamaterial loading reveals a significant key performance modifier. Besides other geometrical parameters like the substrate thickness (***t***), the V arm notch has a considerable impact on the major antenna parameter, as shown in Figure 5a–f. For instance, Figure 5a illustrates the metamaterial DNG unit cell loading effect on both sides of the proposed antenna. The inset of the figure clearly shows that without any meta unit, the loading reflection (S_11_) was not significant between 4.25 and 4.4 GHz. Still, there was a gradual increase in the number of unit cells enhancing the S-parameter from −22 dB to −43 dB in the simulation. The absence of the meta unit or one unit cell does not demonstrate any improvement of S_11_, whereas the arrangement of five units is much better than the rest. As mentioned earlier, the DNG unit cell-balanced structure reinforces the EM field along with the proposed antenna structure. However, a mutual coupling of the surface current reduces and slightly shifts the refection parameter for four unit cells. Hence, incorporating patch geometry is a great effort to improve the performance. Then again, the ground plane with two adjacent splits illustrates a shifting of the resonance frequency as well as broadening the bandwidth. For example, gl = 15 mm shows multiple resonance frequencies between 2.75 and 4 GHz, but gradually, major resonance shifts above 4 GHz as the height increases (Figure 5b). The substrate thickness and notch length variation follow the same trend of resonance shifting. The lower notch length enhances the gain rather than the higher dimension in the expected 4–5 GHz frequency spectrum, as shown in Figure 5c, because the electric field becomes more dominating at the shorter notch. However, an interesting change was observed for the substrate thickness during the simulation. At the 0.5-mm thickness, the proposed retro VMA gives S_11_ exactly at 2.5 GHz with −33 dB as a potential ISM band operation. As ***t*** increases, a smooth tuning of the reflection parameter is demonstrated and operated between 4 and 4.5 GHz. In Figure 5e, the realized gain (dBi) is shown with the slotted notch length changes, with insignificant variation. At the lower and upper band, negative gain is observed, and hence, the notch length was selected to be 4.60 mm since it has an average value of 4 dBi. The axial ratio (AR) bandwidth performance (Figure 5f) to ground plane height shows a range of variation between 17 and 40 dB. Thus, the polarization of the E-field, especially the circularly polarized field, is made up of two orthogonal E-field components of equal amplitude that do not comply with the standard. Further study of the antenna will improve this factor.

### 2.5. Field Analysis and Surface Current

**Figure 6:**
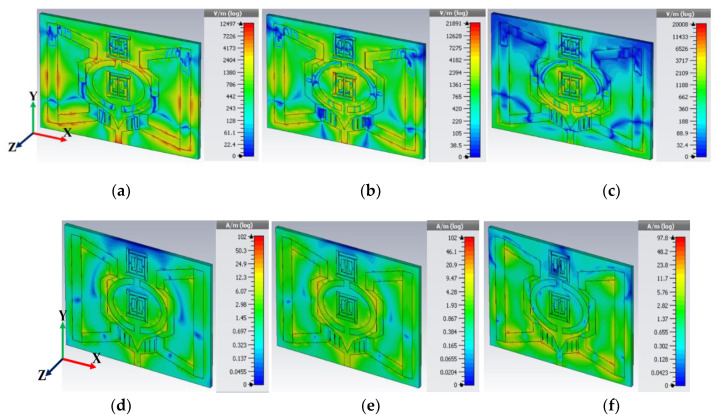
E-field (**a**–**c**) and H-field (**d**–**f**) performance of the proposed antenna at 4, 4.27, and 4.4 GHz (left to right, respectively).

EM field variation is evident for any microstrip patch antenna since the dimensions are finite both in length and width. Hence, the fields at the edges of the patch go through fringing since the proposed retro VMA antenna was modelled using the transmission line principle and are unable to support pure TEM mode. Conventionally, we know the electric field moves in the substrate and even a bit out of the substrate into the air. So, to understand the field variation during EM wave propagation, we must consider the fringing field line as an impact of *ε_reff_* (as per Equation (2)). Since *ε_reff_* is less than the substrate permittivity in terms of the numerical approach, the EM field propagation continues not only in the structure but also in the surrounding air too. So, the field analysis shown in Figure 6 can be analyzed using the basic model (TM_10_). Here, the patch length must be less than λ/2 (wavelength in dielectric medium) and equal to λ_0_/√(*ε_reff_*), where λ_0_ is the free space wavelength used to consider this condition. Thus, the E-field varies and shows an intense electric field concentration by one λ/2 cycle along the X and Y-axis [39]. Starting from 4 GHz (Figure 6a) to 4.4 GHz (Figure 6c), it was observed that the extended slotted arm with split parabolic horns exhibits a strong to moderate electric field concentration, referring to Equation (5) [40]:

(5) $$f_{0}=\frac{\nu_{0}}{2\sqrt{\varepsilon_{reff}}}\left[{\left(\frac{m}{L}\right)}^{2}+{\left(\frac{n}{w}\right)}^{2}\right],$$

where *m* and *n* are the mode of EM wave propagation through the proposed antenna. Other important changes along the length and width are microstrip patch variation, which intensifies the extended arm tank circuits in the two sides’ resonance to exhibit a significant E-field and H-field concentration. The harmonic resonance of the LC tank circuit becomes distorted as the frequency increases and is deformed by field polarization. Remarkably, a minor difference in the magnetic field concentration is noted in Figure 6d–f. From Maxwell’s equation, the intensity of the electric field *E* and magnetic flux density *B* are related using the curl of the electric field [41]. Similarly, the magnetic field intensity *H* and electric flux density *D* use the same calculation with an extra parameter current density *J*. Therefore, the standard ‘Helmholtz equation’ solution comes as a wave vector potential as:

(6) $$E=-j\omega \mu \varepsilon A+\frac{1}{j\omega \varepsilon }\nabla (\nabla .A),$$

(7) H=∇×A,

where *A* is the magnetic vector potential. So, the frequency dependency and vector component are optimized with increasing frequency, whereas the magnetic field intensity only has a vector product of *A*. In the case of UWB antennas, it just concentrates both fields because of the wide conducting patch area. Thus, the S parameter gives a consecutive resonance or wideband resonance spectrum.

**Figure 7:**
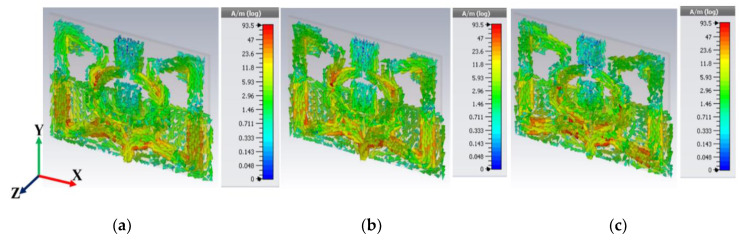
Surface current distribution of the proposed antenna at 4, 4.27, and 4.4 GHz (**a**–**c** respectively).

The surface current distribution (Figure 7) of the low-profile antenna at three distinct frequencies was analyzed to characterize the patch’s key properties. Numerous reported articles have considering various facts like the dielectric layer [42], and key parameter analysis like the radiation pattern, Q factor [43], green function for the wave equation [44], etc. Though limitations in each approach exist, nevertheless, we preferred the green function method since the microstrip patch is thin (z = z′), and the characteristics of the impedance are known. It was assumed that the patch surface current has a three-dimensional component, and the corresponding EM field component can be obtained using Equations (6) and (7). However, using these two equations and applying the green function, the solution obtained for any arbitrary patch [44] is:

(8) $$E_{x,y}=\frac{j\omega }{{\left(2\pi \right)}^{2}k^{2}}\iint_{surface}\varsigma_{surface}e^{jk_{x}k_{y}},$$

where the *k* wave number along *x* and ζ is the tangential electric field component of the corresponding surface current. Hence, the lower frequency surface current of the proposed antenna remains insignificant since the electric field does not show the dominating resonance point at the fabricated structure. At 4 GHz, the surface current near the feedline and extended slotted arm started to show approximately 23.6 A/m(log). The gradual increase of the operating frequency (like 4.27 and 4.4 GHz) continues to show an almost similar current density but loses a significant *E_x_* and *E_y_* field component. Consequently, after 4.4 GHz, the amount of surface current started to decrease.

## 3. Experimental Results and Discussion

**Figure 8:**
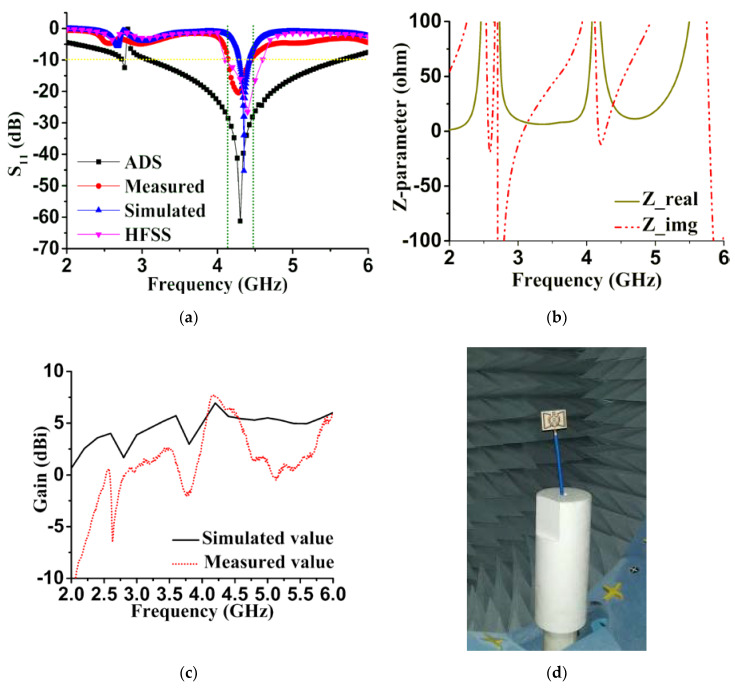
Experimental and simulated results’ comparison; (**a**) Reflection coefficient (S_11_) performance; (**b**) Numerical plotting of characteristic impedance (Z); (**c**) antenna measurement at Sattimo StarLab; (**d**) Gain (dBi) of the proposed antenna.

We further justified the performance of the proposed retro VMA, fabrication, and after the measurement was completed, as shown in Figure 1c and Figure 8c. Comparing the reflection coefficient (S_11_) performance by using the commercially available Advanced Design System (ADS) 2017, CST microwave studio, HFSS, and measurement, good agreement relies on the simulation and measurement. The reflection coefficient measurement was conducted using the keysight Agilent N5227A PNA microwave network analyzer and radiation analysis through Satimo StarLab, Microwave, and the satellite laboratory, UKM. ADS simulation was completed based on the equivalent circuit shown in Figure 2. The S_11_ response from ADS shows quite a good response due to minimal computational constraints and the simulation parameter. Besides, the response was used to get the idea of the equivalent circuit’s compliance with the microstrip patch of the proposed antenna. Figure 8a also reveals that the difference between the simulated and measured data is due to the fabrication tolerance and the resistivity form while connecting the SMA port for measurement. However, the three numerical analyses indicate a resonance frequency at approximately 4.34 GHz with −25 to −60 dB variation. The measured S_11_ is maximum −20 dB at 4.34 GHz with a slightly wider bandwidth of 0.13 GHz. Concerning the factors of patch structure, substrate height (t), and feeding patch, the transmission line model-based characteristics’ impedance follow Equation (9) to match the 50 Ω value [45]:

(9) $$\frac{dZ_{i}}{dx}+j\beta \frac{Z_{i}^{2}}{Z(x)}-j\beta Z(x)=0,$$

where *Z(x)* is the characteristic impedance, *Z_i_* is the input impedance, and *β* is the phase constant. So, Figure 8b represents changes of *Z(x)* by plotting the real and imaginary values. Though the 49.93 Ω real value is the initial value at 4.27 GHz, unexpectedly, it decreases to 30 Ω gradually at 4.4 GHz. In Figure 8d, the simulated and measured gain (dBi) are illustrated as a performance comparison. Even though the negative gain is shown occasionally at some lower and higher frequencies, the expected operational bandwidth was achieved up to 7.14 dBi. The negative portion simply means the S_11_ response became insignificant due to ohmic and dielectric loss of the substrate or coupling of the unit cell antenna and SMA port, external radiation from the feeds and junctions, and excitation of the surface wave.

**Figure 9:**
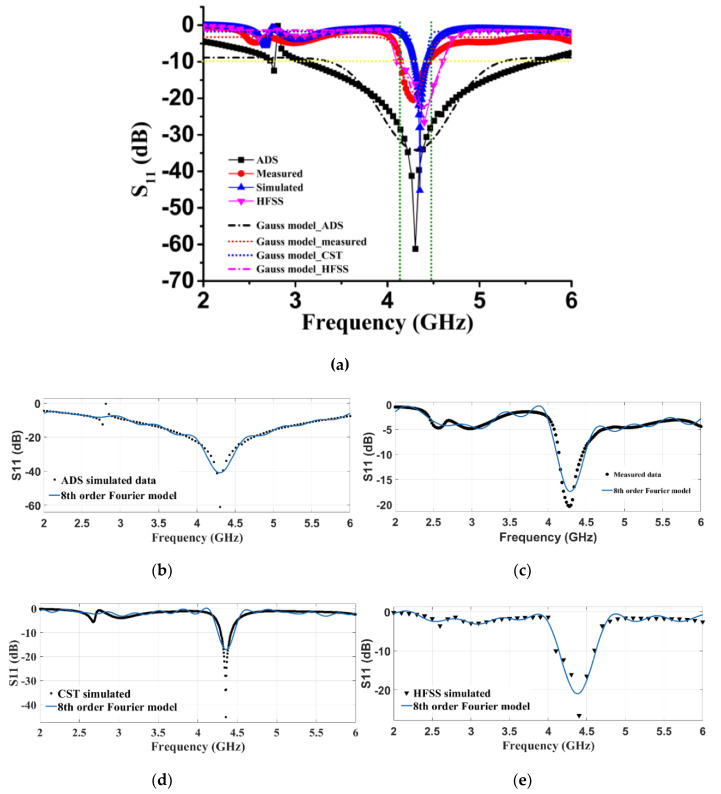
S_11_ Response and data analysis using curve fitting (**a**) Gaussian model; Fourier transform method for (**b**) ADS; (**c**) Measured; (**d**) CST simulated; and (**e**) HFSS simulated.

Two different curve fitting techniques were adopted, namely the Gaussian model and the Fourier Series (FS) model. The Gaussian model general expression [46] is:

(10) $$y=y_{0}+\frac{Ae^{\frac{-4\ln(2){\left(x-x_{c}\right)}^{2}}{w^{2}}}}{w\sqrt{\frac{\pi }{4\ln(2)}}},$$

where *y*_0_ = base, *A* = area, *x_c_* = center, and *w* = FWHM. For each data, a corresponding Gaussian model plotted in Figure 9a represents the fitted curve of ADS, measured, CST, and HFSS-simulated reflection value, respectively. The regression model encountered the fourth-order degree of freedom (DF), and the number of iterations was 9 in the completion of the fitting for each data. The analysis reveals that the mean square weighted deviation (MSWD) or reduced chi-square values for CST simulation and measured are 2.00 and 2.10, respectively. Similarly, the MSWD values for ADS and HFSS are 15.5 and 1.86. Thus, a comparative curve fitting shows the deviation of data between CST and the measured are closer to unity. Therefore, the predicted data deviation of these two methods has a lesser error percentage of 4.6% (for CST) and 11.1% (for measured values).

On the other hand, the FS model converges at the eighth order with the best-fitted data for the four different reflection coefficient (S_11_) data set. Figure 9b–e clearly shows all approximated S_11_ data using the following general model FS function:

(11) $$y=a_{0}+\sum_{i=1}^{8}a_{i}\cos(i\omega x)+b_{i}\sin(i\omega x),$$

where *a*_0_ = model constant, *ω* = fundamental frequency of the signal, and n = 8, the number of harmonics. The adjusted R-square for ADS, measured, CST, and HFSS are 0.9063, 0.9324, 0.7543, and 0.908, respectively. It is an unbiased estimate of the fraction of variance, since it was identified using a precise sample size and some variables. Observing the MATLAB analysis, the FS model of CST simulated is best fitted for ideal S_11_ data prediction. Although the measured data for the FS model are much higher than 0.75, unfortunately, this is the best available approximation in terms of the computation accuracy and practical perspective. So, the reason behind the deviation of the measured and simulated results has a rational explanation. Furthermore, the ADS and HFSS software both have an approximated reflection coefficient either based on the circuit parameter or the simulation set-up. For ADS, each inductor and capacitor (Figure 2) is estimated for the equation as mentioned earlier. Further investigation and tuning of these components may reduce this deviation. In HFSS, tetrahedral meshing and adaptive meshing were performed separately with a limited number of points. It may lead to possible variation of S_11_ in the simulation.

**Figure 10:**
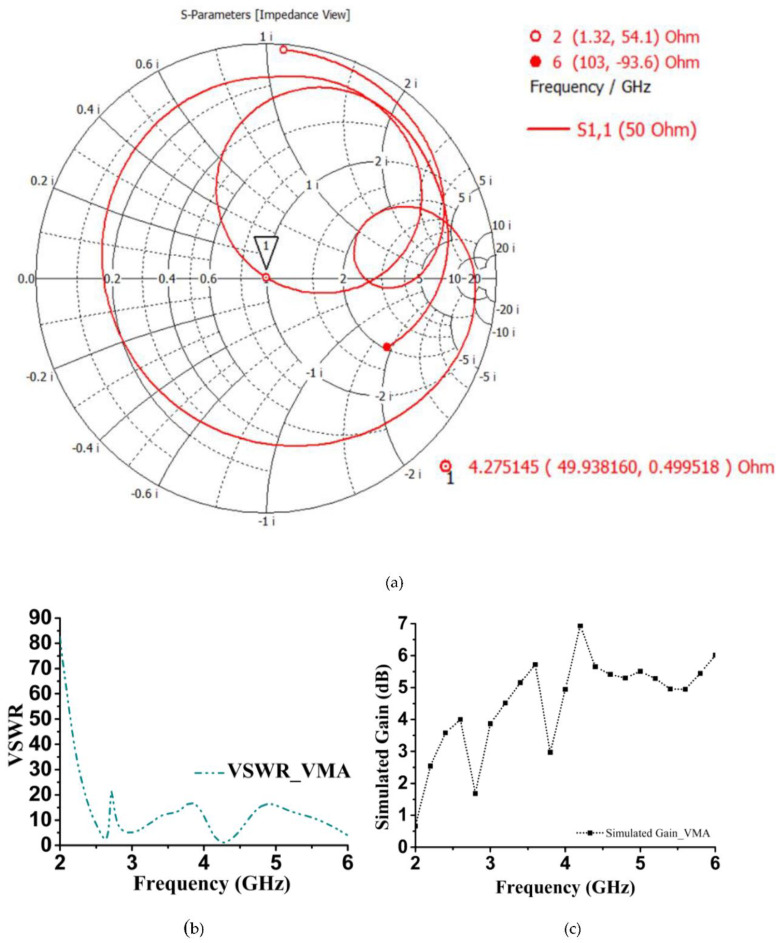
Proposed retro VMA performance; (**a**) Smith chart for impedance characteristics; (**b**) VSWR; and (**c**) simulated gain (dB).

Since the proposed antenna followed the transmission line principle to be designed and developed, the smith chart would be an appropriate tool to analyze the impedance (Z). Figure 10a illustrates Z from 2–6 GHz but particularly focusing on the expected bandwidth operation, which is 4.27–4.4 GHz from the SC (short circuit) to OC (open circuit) terminal. Starting from the high impedance end (at 2 GHz) with two full-wave rotations along the transmission line results in 49.93 Ω at 4.27 GHz. As the maximum measured reflection coefficient point at 4.30 GHz, as shown in Figure 8a, the impedance shifts to 55.05 − j1.8 and continues to reach 33.72 + j16.08 at 4.40 GHz. VSWR (voltage standing wave ratio) of the proposed antenna is approximately the following (Figure 10b) standard value over the operation band. Earlier, the few reported antennas [47,48] recommended, for potential applications of the most slotted shape, values less than 2. The proposed VMA shows a VSWR of 1.01 at 4.27 GHz and was maintained close to that up to 4.34 GHz. After that, it increased to 3 at 4.40 GHz and matching between the transmission line decreased. This compatible VSWR gives a high directivity of more or less 7 dBi. Besides, the simulated gain (dB) also aligns with the claim, as shown in Figure 10c, where the operational bandwidth has a range of 6.93–5.65 dB.

**Figure 11:**
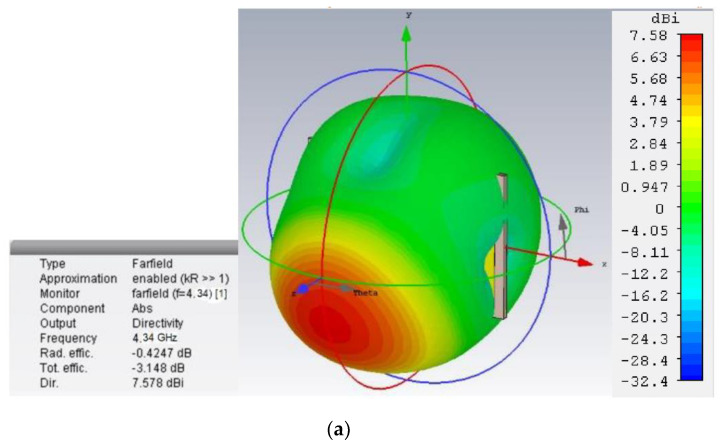

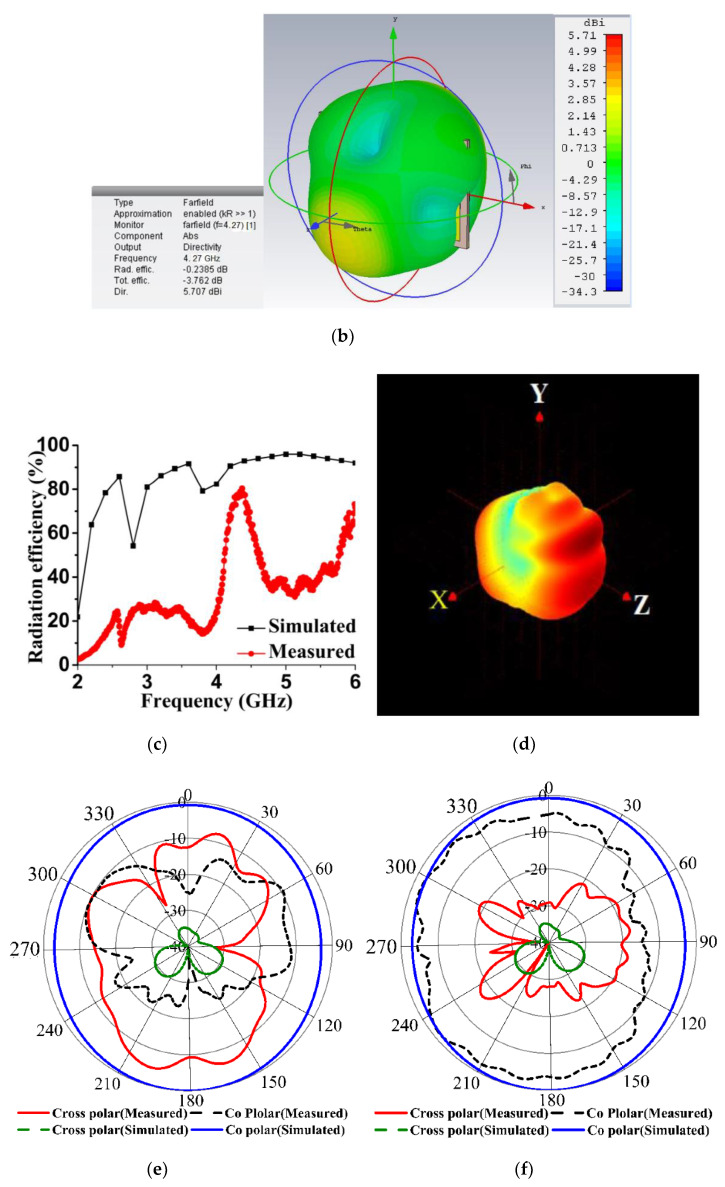
Radiation characteristics of the proposed antenna far-field radiation pattern (**a**) at 4.34 GHz; (**b**) 4.27 GHz; (**c**) Simulated vs. measured radiation efficiency; (**d**) Measured radiation at 4.30 GHz; (**e**) E-plane radiation; (**f**) H-plane radiation at 4.30 GHz.

Figure 11a,b shows the simulated 3-D far-field radiation pattern of the proposed antenna, where the radiation efficiency is about 90% at 4.34 and 4.27 GHz. However, the total efficiency is reduced by 46% and 38%. Furthermore, the simulated and measured radiation efficiency are plotted in Figure 11c. The experimental efficiency dropped down to 80% compared to the simulated one. However, beyond and above the stated bandwidth, this difference is much more. A lack of proper finishing during etching leads to the copper layer being missed (less than 0.1 mm in size) in two vertical extended arm edges. Therefore, it may affect the radiation efficiency. A measured radiation pattern at 4.30 GHz is illustrated in Figure 11d, which was measured in Satimo Starlab, UKM. The radiation characteristics of the E-plane (Figure 11e) and H-plane (Figure 11f) were compared through a simulation and measured environment at 4.30 GHz. The co-polarization and cross-polarization have some deviation due to an unequal phase distribution, but referring to the specific directional operation, it has strong field pattern compliance. However, a general discussion of the out-of-band rejection of the prototype introduces two issues. First, the proposed antenna structure and microstrip patch followed the narrow adjacent line rather than the wider arm formation. Furthermore, the antenna must maintain the minimal dimension for a low profile, so the effect of reducing the substrate thickness reveals the tunability property. Secondly, the electric field at 4.27 GHz indicates an extended V arm has a more concentrated distribution in several horizontal, vertical, and tapered patches but increasing the resonance frequency loses this field. Besides, it concentrates the field closer to the feeding point precisely on the lower part of the parabolic horns. Hence, the out-of-band peaks are a combination of the two effects above, and both can be tuned by the substrate permittivity value. This tuning is limited by two reasons: (1) On the one hand, the change of permittivity modifies the width of the antenna; and (2) the values of dielectric permittivity are conditioned to the available commercial substrates. A comparison of the features is tabulated in Table 2 in terms of the design gain and development technique, physical geometry, etc. for understanding the contribution of the proposed retro VMA. Certainly, the low-profile antenna with a thin microstrip patch is unable to meet a few novel characteristics. For example, an incompatible dimension due to the application perspective, conformality, radiation efficiency with specific gain, and polarization insensitivity. A few relevant papers have a relatively low and high dimension with competitive parameters. Still, they are underwhelming in terms of the dimension constraint, efficiency, or adopted technique limitations for the prospective application field. For example, the transmission line model [49] or filtering [14] technique has a lower dimension compared to the proposed antenna. Still, those prototypes have limitations like a reduced realized gain or are bulky in size. Similarly, the characteristic model [50] and the split ring method [51] either suffers from impedance accuracy of the overall structure or a lack of experimental validation of the projected data. Besides, a robust design approach, such as stacking [52] or frequency selective surface (FSS) [53], has a significant realized gain, though the efficiency was not reported in the corresponding article.

Moreover, the dimension of these antennas would be incompatible in some 5G applications like IoT and smart devices. The proposed antenna exhibits potentially better key performance parameters, such as the measured realized gain of 7.14 dBi and efficiency of 80%. In contrast, the nearest competitive prototype is above 82% but is unable to maintain the standard characteristic impedance in the operating frequency.

## 4. Conclusions

A slotted loaded with DNG metamaterial antenna was proposed for 5G applications. By combining the meta unit cell’s inherited dielectric characteristics’ modification and the extended arm structure, a low profile and narrowband directive features were achieved. The physical geometry was shown to provide balanced EM resonance to maintain standard VSWR and a maximum 7.14 dBi gain at 4.34 GHz. Furthermore, the electric field and magnetic field distribution on the corresponding bandwidth ensure a constant operating frequency for future 5G use case scenarios like smart home applications, IoT, and other features.

## Figures and Tables

**Table 1:** The dimensions of proposed retro VMA.

Parameter	a	b	c	d	e	f	g	h	i
Value (mm)	60	40	35.20	15.72	5.60	11.20	7.12	4.66	5.01
Parameter	j	k	l	m	n	o	p	q	gl
Value (mm)	17.52	24.80	11.19	5.30	4.32	2.40	14.50	1.0	20

**Table 2:** Performance comparison of the proposed antenna with relevant reported antennas.

Reference	Design Technique	Dimension (mm)	Operating Frequency Band (GHz)	Maximum Realized Gain (dBi)	Efficiency (%)	Remarks
[50]	Characteristic model	20 × 20 × 1.6	5–20	9	82–87	Superstrate metamaterial used for performance enhancement
[49]	CRLH-TL method	30 × 30 × 1.6	0–10	5.2	78	Monopole antenna
[52]	Stacking	180 × 60	0.5–4	6.1	NR	5G lower frequency
[51]	Split Ring method	40 × 45 × 1.57	3–4	7.43	NR	No experimental evaluation
[14]	Filtering	40 × 40	3–5	9	NR	Bulky in size
[53]	FSS	134.5 × 178.14 × 177	3–10	13.9	NR	Bulky in size
Proposed antenna	DNG metamaterial loaded	60 × 40 × 1.52	2–5	7.14	80	Low profile and directive

NR = Not reported.
